# Radiomics-based aortic flow profile characterization with 4D phase-contrast MRI

**DOI:** 10.3389/fcvm.2023.1102502

**Published:** 2023-04-03

**Authors:** Markus Huellebrand, Lina Jarmatz, Chiara Manini, Ann Laube, Matthias Ivantsits, Jeanette Schulz-Menger, Sarah Nordmeyer, Andreas Harloff, Jochen Hansmann, Sebastian Kelle, Anja Hennemuth

**Affiliations:** ^1^ Deutsches Herzzentrum der Charité, Berlin, Germany; ^2^ Charité – Universitätsmedizin Berlin, corporate member of Freie Universität Berlin and Humboldt Universität zu Berlin, Berlin, Germany; ^3^ Fraunhofer Institute for Digital Medicine MEVIS, Berlin, Germany; ^4^ Charité Universitätsmedizin Berlin, Working Group on Cardiovascular Magnetic Resonance, Experimental and Clinical Research Center, a joint cooperation between the Charité Universitätsmedizin Berlin and the Max-Delbrück-Center for Molecular Medicine, Berlin, Germany; ^5^ DZHK (German Centre for Cardiovascular Research), partner site Berlin, Berlin, Germany; ^6^ Helios Hospital Berlin-Buch, Department of Cardiology and Nephrology, Berlin, Germany; ^7^ Department of Neurology, University Medical Center Freiburg, Freiburg, Germany; ^8^ Faculty of Medicine, University of Freiburg, Freiburg, Germany; ^9^ Department of Radiology, Theresienkrankenhaus und St. Hedwig-Klinik, Mannheim, Germany; ^10^ Department of Diagnostic and Interventional Radiology and Nuclear Medicine, University Medical Center Hamburg-Eppendorf, Hamburg, Germany

**Keywords:** Radiomics, 4D PC-MRI, flow profile, aortic valve stenosis, population, travelling volunteers, reproduciblity

## Abstract

4D PC MRI of the aorta has become a routinely available examination, and a multitude of single parameters have been suggested for the quantitative assessment of relevant flow features for clinical studies and diagnosis. However, clinically applicable assessment of complex flow patterns is still challenging. We present a concept for applying radiomics for the quantitative characterization of flow patterns in the aorta. To this end, we derive cross-sectional scalar parameter maps related to parameters suggested in literature such as throughflow, flow direction, vorticity, and normalized helicity. Derived radiomics features are selected with regard to their inter-scanner and inter-observer reproducibility, as well as their performance in the differentiation of sex-, age- and disease-related flow properties. The reproducible features were tested on user-selected examples with respect to their suitability for characterizing flow profile types. In future work, such signatures could be applied for quantitative flow assessment in clinical studies or disease phenotyping.

## 1. Introduction

Four dimensional velocity encoded phase-contrast MRI (4D PC MRI) provides volumetric time-resolved velocity measurements in three spatial directions (1). Clinical interpretation of the highly complex information in the context of aortic diseases has been approached in several studies, which explored the association of flow patterns with pathologies of the aortic valve and vessel wall (2). In many studies, flow patterns were assessed qualitatively based on the inspection of vector visualizations, streamlines, and pathlines (3). Quantitative approaches for the assessment of local hemodynamics are measuring local properties of the velocity vector field in vessel segments or cross-sections (4–6). Helicity- and vorticity-based measures characterize local circulatory flow independent of orientation (7, 8). Other parameters provide information of flow properties with regard to the vascular anatomy. The normalized flow displacement and the angle have been established as measures of flow eccentricity (9–11). Schafstedde et al. (12) have introduced the wall parallelity degree (WPD), defined as the ratio between flow through the vessel cross-section and total flow, to quantify the part of blood flow parallel to the vessel centerline. These measurements use the cross-sectional vessel segmentation to derive the vessel center and the local vessel orientation. The wall shear stress (WSS) has been suggested for the assessment of the impact of flow phenomena on the vessel wall (11, 13). The calculation of this parameter requires the vessel surface points and the local orientation (surface normals) of the vessel wall (14). For the assessment of reflow phenomena, Rodriguez-Palomares et al. further considered the systolic flow reversal ratio (15). The quantitative flow profile analysis using the parameters mentioned above is typically based on aggregating the values per cross-section. We found only one publication which considered flow parameter texture: Garcia et al. reported significant differences in skewness and kurtosis of cross-sectional velocity distributions depending on age, sex and disease (16).

The reproducibility of conventional cross-sectional aortic flow quantification in 4D PC MRI with regard to acquisition, scanner, and observer interaction has been analyzed in previous publications. Morphometric parameters of cross-sections and vessel segments have been analyzed with excellent to good reproducibility for repeated scans, repeated analyses, and different observers in volunteers at a single scanner (17). The reproducibility of wall shear stress and velocity measurements was still moderate to good for systolic phases (18). The recent comparison of flow and wall shear stress measurements between acquisitions with scanners from different manufacturers showed considerable differences (19). The best agreement was found at the sinotubular junction.

Radiomics features have become popular for assessing morphology and tissue properties of anatomical structures (20). Shape characteristics are quantified through features such as area, elongation, and compactness, while co-occurrences and differences in neighboring voxel intensities allow the assessment of intensity patterns. The Image Biomarker Standardization Initiative (ISBI) has defined a set of radiomics features in such a way that implementing software packages now provide reproducible results (21). While radiomics features have been successfully used for phenotyping in oncology, their potential in the area of flow characterization has not yet been explored. As radiomics features depend on the underlying segmentation and the voxel intensity values, they offer a joint assessment of vessel shape and blood flow velocity in the proposed context of 4D PC MRI.

The purpose of this work is to evaluate a radiomics approach for the characterization of flow profiles to enable quantitative phenotyping. To this end, we analyze the reproducibility of radiomics features and study their applicability to the differentiation of normal and pathological flow profiles in the ascending aorta.

## 2. Materials and methods

**Figure 1:**
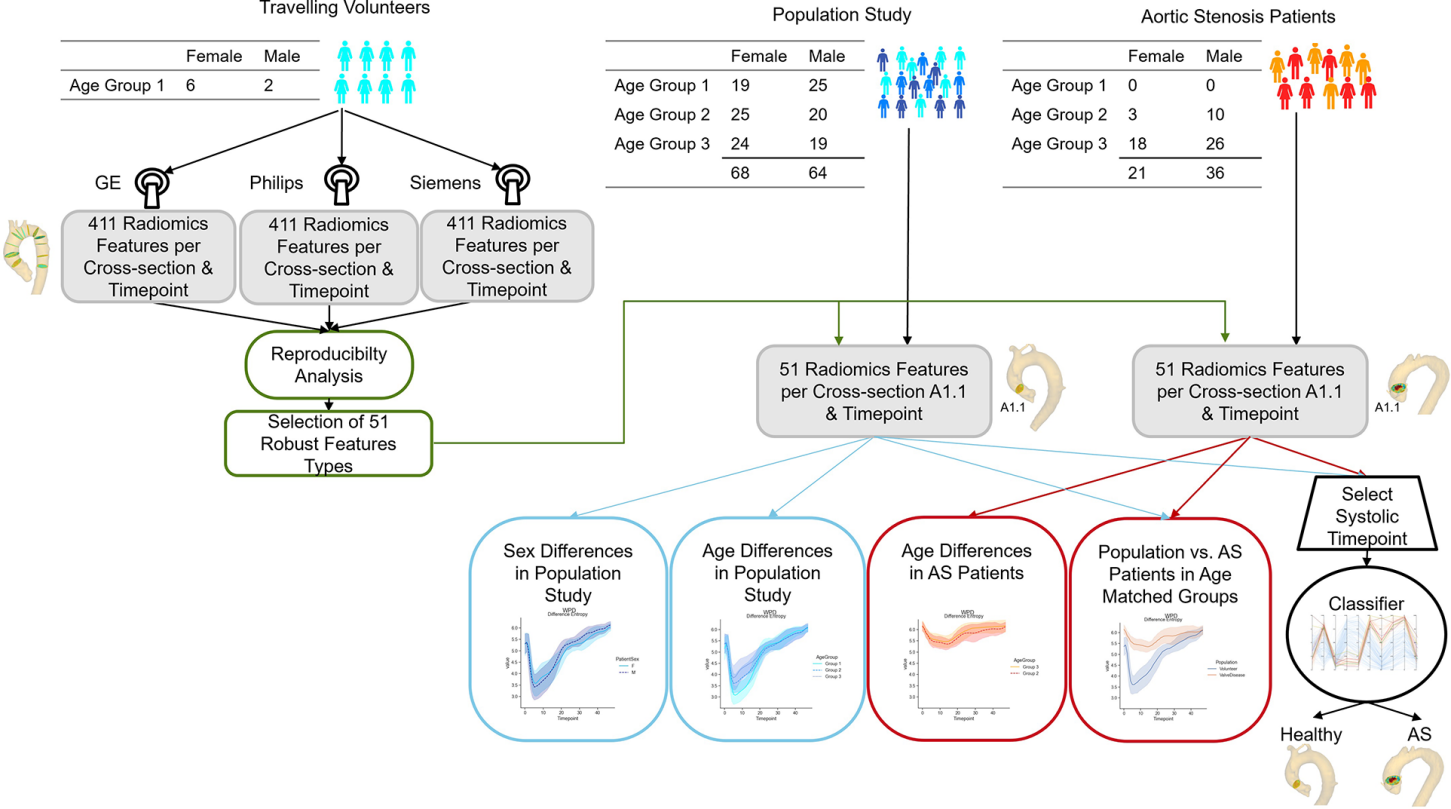
Flow chart of the presented study. Image data from eight volunteers acquired with GE, Philips and Siemens scanners are used to assess the reproducibility of 411 radiomics features extracted on twelve imaging planes across the thoracic aorta throughout the cardiac cycle. The 51 most robust features are extracted for age- and sex- stratified cohorts of aortic stenosis patients and subjects without valve diseases for comparison. Age-matched cohorts are used to assess whether differences in flow profile are identifiable in the radiomics signatures. Finally, a classifier to differentiate healthy subjects from aortic stenosis patients is trained on the radiomics features of a cross-section in the ascending aorta (A1.1) for a peak systolic timeframe.

We investigate radiomics shape features as well as texture features of scalar flow quantities, such as the through-plane flow, the angle between flow vector and cross-section normal, and normalized helicity and vorticity. We analyze the reproducibility of these features with respect to acquisition and observer. Furthermore, we use two age-matched cohorts to test whether the reproducible features are suitable for distinguishing different types of flow profiles. A flow chart of the proposed analyses is given in Figure 1

### 2.1. Data

Our analyses use data sets from three independent studies (Table 1). For the reproducibility assessment of the feature calculation, we consider eight healthy subjects from the travelling volunteer study presented by Demir et al. (19). Each volunteer was scanned on Philips (Ingenia 3T), Siemens (Prisma Fit) and GE (SIGNA Architect 3T) systems with the scan parameters as summarized in Table 1.

**Table 1:** Overview of the demographic and imaging properties of datasets used for the reproducibility (travelling volunteers) and applicability analyses (aortic stenosis patients and population study).

	Travelling volunteers			Aortic stenosis patients	Population study
Subjects	8			57	132
Age (years)	25±6			66±10	50±17
Weight (kg)	60±10			81±14	76±18
Female	6 (75%)			22 (42%)	68 (52%)
Scanner	Philips Ingenia	Siemens Prisma Fit	GE SIGNA Architect	Philips Achieva	Siemens TrioTim
Field Strength	3T	3T	3T	1.5 T	3T
Velocity Enc. (cm/s)	250	150	150	350–600	150
ECG gating	Retrospective	Prospective	Retrospective	Retrospective	Prospective
Repetition time (ms)	3.5	5.1	4.2	3.5	5
Echo time (ms)	2.2	2.6	2.00	2.2	2.54
Flip angle (${}^{\circ }$)	5	8	8	7	7
Voxel size (mm${}^{3}$)	2.8×2.8×2.8	2.7×2.3×2.6	2.4×2.4×2.8	2.5×2.5×2.5	2.5×2.1×2.5
Timeframes/cycle	25.00	20.00	25.62±0.52	25.00	37.93±5.81
Temp. res. (ms)	28.00	40.8	66.8	38	20
Heart rate (bpm)	75.00±5.58	59.21±8.59	77.12±5.06	65.48±8.38	65.23±13.26

The applicability of radiomics analysis to characterize flow profiles is tested with data from two additional independent studies. A dataset of patients with aortic stenosis (22) represents a cohort of pathological cases, while a dataset from a population study in the city of Freiburg, Germany (23) serves as control. We were able to include six additional cases in the latter dataset, which could not be analyzed in the initial study. None of the control subjects were diagnosed with valve diseases.

### 2.2. Image analysis and flow profile assessment

**Figure 2:**
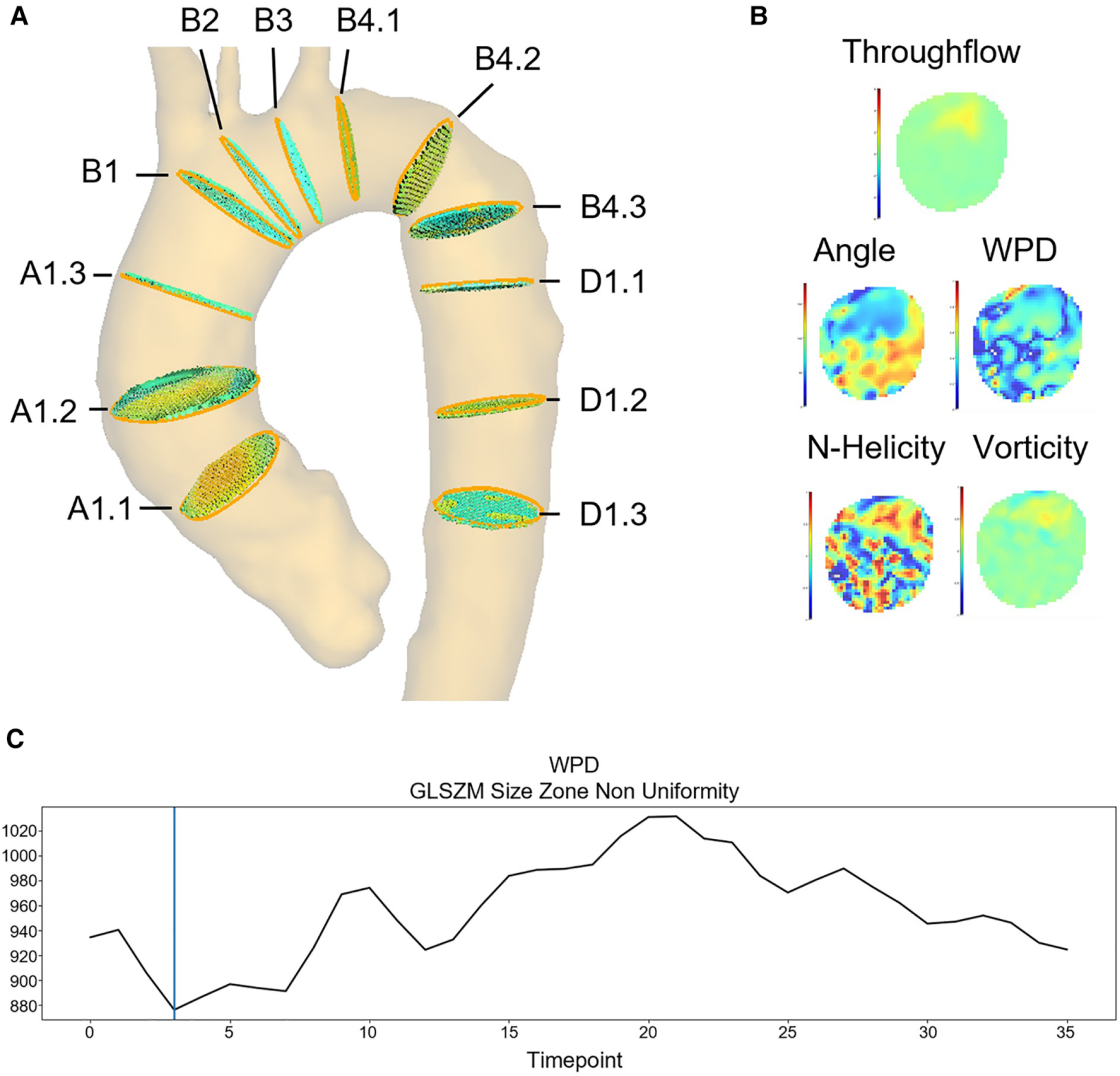
Feature extraction example: (**A**) 12 standardized image planes covering the ascending aorta, the aortic arch and the descending aorta. (**B**) Flow parameter maps calculated for each segmented image plane and timeframe (here plane A1.1): throughflow, angle, WPD, LNH and vorticity. (**C**) Feature curve showing the GLSZM Non-Uniformity based on the WPD map throughout the cardiac cycle on plane A1.1.


4D flow MR images are analyzed using MEVISFlow. Preprocessing includes background phase offset correction with a polynomial fit and phase unwrapping with the PRELUDE algorithm. The aortic centerline is calculated via skeletonization of the 3D aorta segmentation in the phase contrast magnetic resonance angiography. To achieve comparability, cross-sections are placed according to the definition in Schafstedde et al. (12) (Figure 2). Observers position four cross-sections along the course of the aorta: distal to the coronary ostia (A1.1), proximal to the branch of the brachiocephalic trunc (B1), behind the subclavian artery (B4.1), and next to the pulmonary artery (D1.1). Additional cross-sections are placed automatically with equidistant spacing within the ascending and descending aorta segments. Cross-sectional region of interest (ROI) are segmented manually by the observer. For each cross-section, the ROI is transferred to all time frames of the image sequence by automatic motion tracking using the morphon algorithm (24). The cross-section-based multiplanar reconstructions are resampled to a voxel resolution of 1 mm${}^{3}$ using trilinear interpolation. For the radiomics analysis, parameter maps are calculated per cross-section and image timepoint (see Figure 2). For the throughflow map, the scalar product of the velocity vector $\vec{v_{i}}$ and the normal $\vec{n_{p}}$ of cross-sectional plane p is calculated for each voxel i∈p (Equation 1).

(1) $$u_{i}=\vec{v_{i}}\cdot \vec{n}$$

For the wall parallelity degree (WPD) map, the throughflow magnitude $u_{i}$ is divided by the total flow magnitude $\left|\vec{v_{i}}\right|$ (Equation 2).

(2) $${\text{WPD}}_{i}=\frac{u_{i}}{\left|\vec{v_{i}}\right|}$$

The angle $\alpha_{i}$ is defined as the angular deviation of the velocity vector $\vec{v_{i}}$ from the plane normal $\vec{n_{p}}$ (Equation 3).

(3) $$\alpha_{i}=\cos^{-1}\left(\frac{\vec{v_{i}}}{\left|\vec{v_{i}}\right|}\cdot \vec{n}\right)$$

Throughflow, wall parallelity degree and angle assess aspects of the flow orientation in relation to the aortic anatomy, which is represented by the orientation and outer contour of the aortic cross-section. The vorticity is defined as the curl of the velocity vector field (equation 4).

(4) $$\vec{\omega_{i}}=\nabla_{i}\times \vec{v_{i}}$$

The absolute local normalized helicity (LNH${}_{i}$) is the normalized dot product of the vorticity $\omega_{i}$ and the velocity vector $\vec{v_{i}}$.

(5) $${\text{LNH}}_{i}=\frac{\vec{\omega_{i}}\cdot \vec{v_{i}}}{\left|\vec{\omega_{i}}|\cdot |\vec{v_{i}}\right|}$$

These parameters describe local flow properties derived from the voxel neighborhood without considering the anatomy.

Unsigned parameters are normalized to the interval [0,4096], signed parameters to [−2048,2047]. The parameter-specific details are shown in Table 2. For each of the five parameter maps, 79 first- and higher-order radiomics features were extracted using the pyradiomics library.^1^ We use the standard fixed bin size suggested by pyradiomics for discretization. With additional 2D shape features, a total of 411 features are extracted on each plane for each measured timeframe. The temporal evolution of each feature throughout the cardiac cycle can be visualized as a feature curve (example in Figure 2).

**Table 2:** Radiomics features were extracted with a fixed bin width 25 (default setting of the pyradiomics library). Each feature map had to be rescaled to enable a robust extraction of the parameters. Input value ranges of the throughflow, angle, wall parallelity degree, normalized helicity and vorticity feature maps are given in the first row. They are mapped to the output ranges shown in the second row.

	Throughflow (m/s)	Angle (${}^{\circ }$)	Wall parallelity degree	N-Helicity	Vorticity
u	α	WPD	LNH	ω	
Input range	[−6,6]	[−0,180]	[0,1]	[−1,1]	[−1,1]
Output range	[−2048,2047]	[0,4096]	[0,4096]	[−2048,2047]	[−2048,2047]

For inter-scanner reproducibility assessment, cross-sections from all aortic segments and cardiac phases are considered. To allow comparison at the highest temporal resolution, the feature curves of sequences with lower temporal resolution are upsampled with linear interpolation. For the inter-observer reproducibility assessment, a second observer processed the eight volunteer datasets acquired with scanner one. For the assessment of the inter-observer and inter-scanner agreement, we calculate the intra-class correlation coefficient (ICC). As suggested in (25), ICC<0.5 is interpreted as poor, ICC 0.5–0.75 as moderate, 0.75–0.9 as good, and an ICC>0.9 as excellent reproducibility. The features with moderate or better inter-scanner and inter-observer ICC are selected for the application tests.

test the applicability of the suggested flow profile characterization, we compare the feature curves of the first ascending aorta plane (A1.1) obtained in the aortic stenosis patients (cohort 2) to those from subjects of the population study without valve disease (cohort 3). We use the set of features found to be reproducible, which describe the distribution patterns of flow directions, contour shape and mobility, as well as features describing the patterns of vorticity and normalized helicity. Subjects are stratified by sex and age group (Group 1: [20–39] years, Group 2: [40–59] years, Group 3: [60–80] years). As for the reproducibility assessment, the feature curves of the sequences acquired with a lower temporal resolution are upsampled to match the sampling of the sequence with the highest temporal resolution. The suitability for disease classification is tested with a logistic regression model trained with the selected radiomics feature set to distinguish between aortic stenosis and no valve disease. Finally, we explore the systolic radiomics signatures of the different datasets and examine representative flow profiles.

## 3. Results

All datasets were successfully processed and considered in the radiomics feature evaluation as displayed in Figure 1.

### 3.1. Reproducibility of radiomics features of flow profiles

#### 3.1.1. Inter-observer reproducibility

The ICC values showed an excellent inter-observer agreement in most features (Excellent: 283, Good: 84, Moderate: 39, Poor: 5, see Table 3), provides an overview of the features for the different parameter maps. Detailed information on the ICC values per feature are given in the supplementary material.

**Table 3:** Number of features grouped by inter-observer agreement and underlying parameter map and contour shape feature type. Reproducibility thresholds were adopted from Koo and Li (25). The majority of the features show an excellent or good agreement for all parameter maps.

Parameter map	Throughflow	WPD	Angle	N-Helicity	Vorticity	Shape
Excellent	53	65	65	42	47	11
Good	20	13	12	17	21	1
Moderate	3	1	2	19	11	3
Poor	3	0	0	1	0	1
Total features	79	79	79	79	79	16

#### 3.1.2. Inter-scanner reproducibility

The ICC values for the inter-scanner agreement are moderate for 51 features and poor for the other 360 features (Table 4). Table 5 shows the top 5 features with moderate agreement per parameter map. Detailed information on the ICC values are given in the supplement.

**Table 4:** Number of features grouped by inter-scanner agreement and underlying parameter map and contour shape feature type. Reproducibility thresholds were adopted from Koo and Li (25). The majority of the features show moderate or poor agreement for all feature types. For throughflow velocities, only one feature showed moderate reproducibility.

Parameter map	Throughflow	WPD	Angle	N-Helicity	Vorticity	Shape
Excellent	0	0	0	0	0	0
Good	0	0	0	0	0	0
Moderate	1	4	26	4	5	11
Poor	78	75	53	75	74	5
Total features	79	79	79	79	79	16

**Table 5:** Inter-scanner agreement (ICC) of all features grouped by type of feature map and contour shape feature type. The best features per parameter map with moderate agreement are listed in the table.

Throughflow	WPD	Angle	N-Helicity	Vorticity	Shape
Gray level non-uniformity (GLRLM)	Inverse variance (GLCM)	Zone percentage (GLSZM)	Run length non-uniformity (GLRLM)	Dependence non-uniformity (GLDM)	Perimeter
	Dependence non-uniformity (GLDM)	Inverse difference normalized (GLCM)	Size zone non-uniformity (GLSZM)	Gray level non-uniformity (GLRLM)	Maximum diameter
	Size zone non-uniformity (GLSZM)	Size-zone non-uniformity (GLSZM)	Dependence non-uniformity (GLDM)	Run entropy (GLRLM)	Major axis length
	Inverse difference (GLCM)	Dependence non-uniformity (GLDM)	Dependence entropy (GLDM)	Coarseness (NGTDM)	Perimeter to surface ratio
	IDM (GLCM)	Inverse difference (GLCM)		90th percentile (first-order)	Area

For the throughflow parameter maps, only the Gray Level Non-Uniformity derived from the Gray Level Run Length Matrix showed moderate agreement between the different scanners. This feature quantifies the similarity of throughflow values and is thus independent of minor differences in the absolute values. Except for 90th percentile of the vorticity values, only higher-order features showed moderate agreement for the parameter map analysis.

Dependence Non-Uniformity derived from the Gray Level Dependency Matrix (GLDM) showed moderate agreement for all maps except for throughflow. The Gray Level Dependence Matrix counts the number of occurrences of homogeneous regions with certain sizes. The Dependence Non-Uniformity measures the similarity of dependence, and a low value means fewer zones, indicating larger homogeneous regions. The last row in Figure 6 shows example vorticity maps and corresponding feature maps for three different cases in a systolic timeframe. Vorticity differences result in higher values while the values are low in homogeneous regions. Features such as the Inverse Variance and the Inverse Difference describe the local homogeneity of the parameter values based on the co-occurrence of neighboring parameter values (GLCM). The second row in Figure 6 shows the Inverse Variance feature for WPD maps. The examples show that the feature values are high where the change in the wall parallelity degree is low. If the Inverse Variance and Inverse Difference values of the throughflow-related parameter values (WPD, Angle) are high, the flow profile is smooth with continuous changes in all neighborhoods. The Gray Level Size Zone Matrix (GLSZM) contains the numbers of connected regions with homogeneous values. The Size Zone-Non-Uniformity measures the variability of size zone volumes and is low in the case of homogeneity. The Zone Percentage measures the coarseness and as displayed in row 3 of Figure 6, a high value indicates many regional changes. If the Zone Percentage is high for the throughflow-related parameters, the flow profile is rough or bumpy. For the vorticity map, parameters describing the heterogeneity of the texture patterns (Run Entropy) via the Gray Level Run Length Matrix (GLRLM) and the Coarseness defined via the parameter deviation from a neighbourhood average (derived from the Neighbouring Gray Tone Difference Matrix (NGTDM)) also show moderate agreement. The Gray Level Run Length Matrices contain the numbers of voxels with similar parameter values per direction. These matrices are then combined. The derived Non-Uniformity features show high values for irregular patterns. For the helicity and vorticity maps this could indicate turbulent flow or noise-affected vector fields. The Neighbouring Gray Tone Difference Matrix quantifies the difference between a value and the average value of its neighbours within a certain distance. A high Coarseness value derived from the NGTDM indicates strong value differences in the defined neighborhood. High values of this feature for the normalized helicity or vorticity indicate small turbulences or noise-affected vector fields.

### 3.2. Application to different age, sex and disease groups

**Figure 3:**
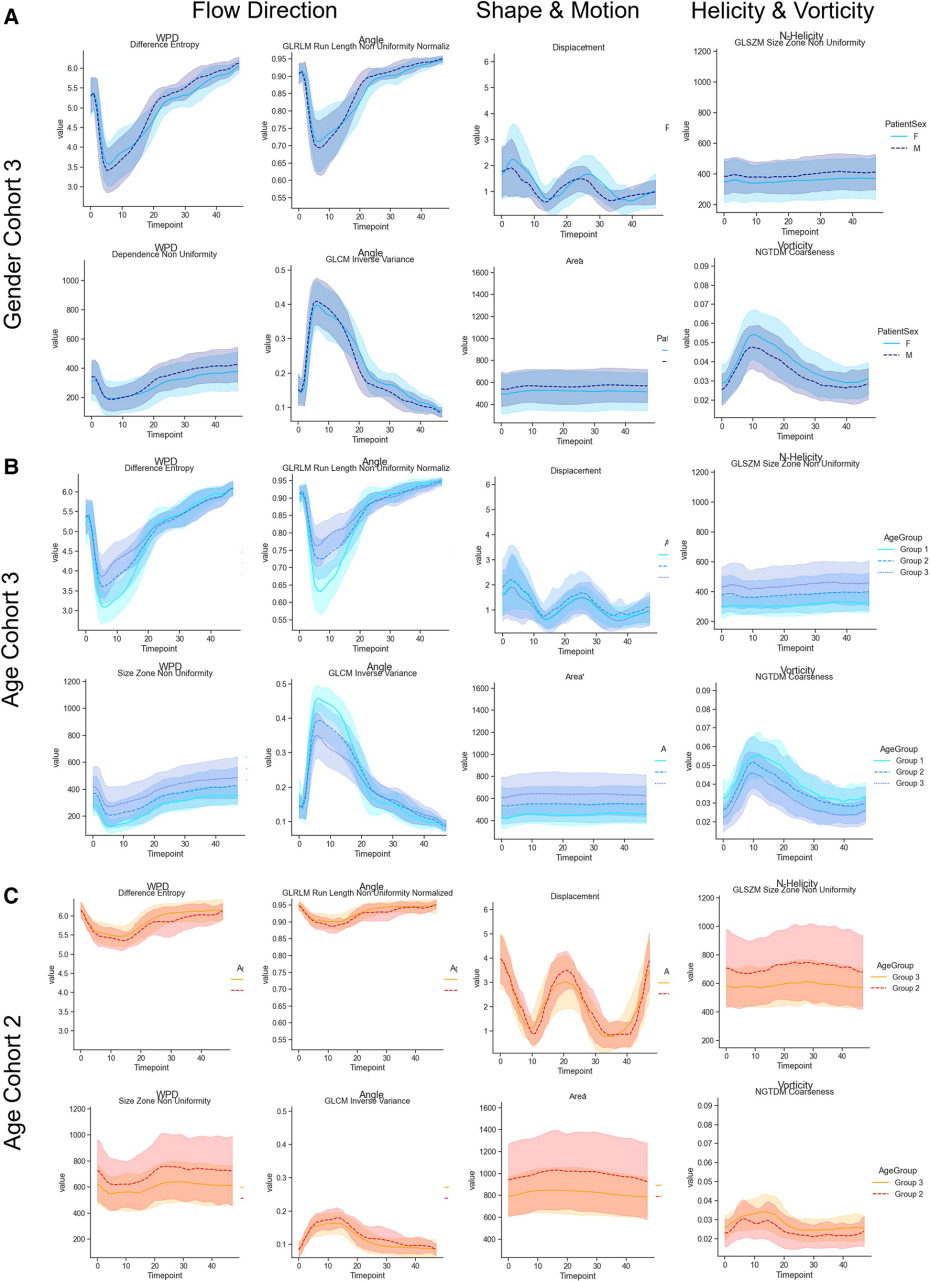
(**A**) Comparison of radiomics feature value ranges in male and female in cohort 3. (**B**) Comparison of radiomics feature value ranges for different age groups for cohort 3 (no valve disease). The curves represent age groups 1–3. (**C**) Shows the curves of age groups 2 and 3 for cohort 2 (aortic valve stenosis). The selected features describe patterns related to the flow direction, shape and motion, and normalized helicity and vorticity-related patterns over the complete cardiac cycle.

**Figure 4:**
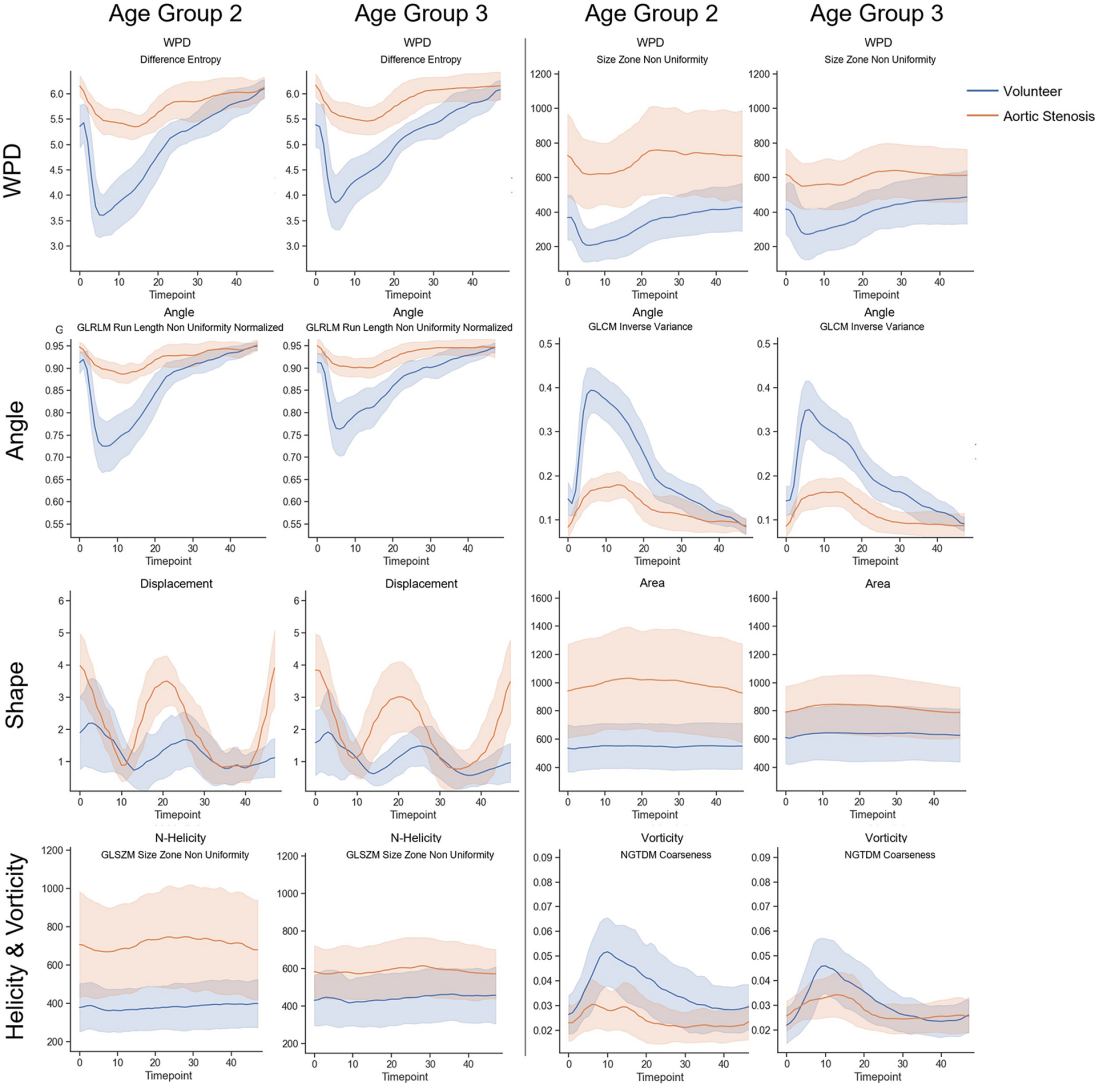
Comparison of radiomics feature value ranges for patients with aortic valve stenosis (cohort2: orange) and age-matched subjects from the population without valve diseases (cohort3: blue). The curves in row 1 show radiomics parameters extracted on WPD parameter maps over the complete cardiac cycle in age group 2 (40–59), and and in age group 3 (60–80). Together with with the parameters shown in row two, which were extracted from flow angle maps, these selected features describe patterns related to the flow direction. The parameters in row three describe the shape and motion, while the parameters in row four describe patterns related to normalized helicity and vorticity-related patterns over.

We selected the features with at least moderate reproducibility with regard to ICC values to test whether they can be used to characterize flow profile differences between age groups and between normal and pathological cases. For an age-matched comparison, the second and third cohorts were separated into three groups (Group 1: [20–39], Group 2: [40–59], Group 3: [60–80]) as defined in (26). and age group feature curves for cohort 3, as well as the age group feature curves for cohort 2 are displayed in Figure 3. On average, the observed cross-sectional area was smaller in women, and there are observable differences in the normalized helicity and vorticity patterns. Age-related trends can be observed in cohort 3 with no valve disease. Age-related differences are less pronounced in aortic stenosis patients. Figure 4 shows the average, minimum and maximum values of features related to the flow direction, shape and motion, as well as vorticity and normalized helicity for the datasets of age groups 2 and 3 in cohorts 2 and 3. The curves of the different cohorts differ in value ranges and curve shapes for both age groups. Shape and motion-related features vary more in age group 2.

**Figure 5:**
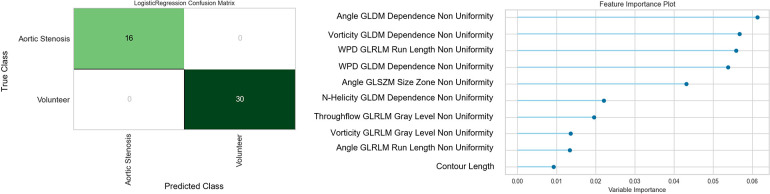
Confusion matrix and feature importance analysis for the logistic regression classifier trained for the separation of flow profiles into aortic valve stenosis vs. normal valves The most important features describe the patchiness of the flow angle, normalized helicity and wall parallelity degree.

The logistic regression model trained on the 51 selected features extracted on the systolic feature maps on plane A1.1 achieves an accuracy and F1-value of 1.0 in the differentiation of cases with aortic stenosis and no valve disease. The confusion matrix and the feature importance analysis are displayed in Figure 5. The features found most important for the classification describe the texture patterns of flow angle, normalized helicity and wall parallelity degree.

**Figure 6:**
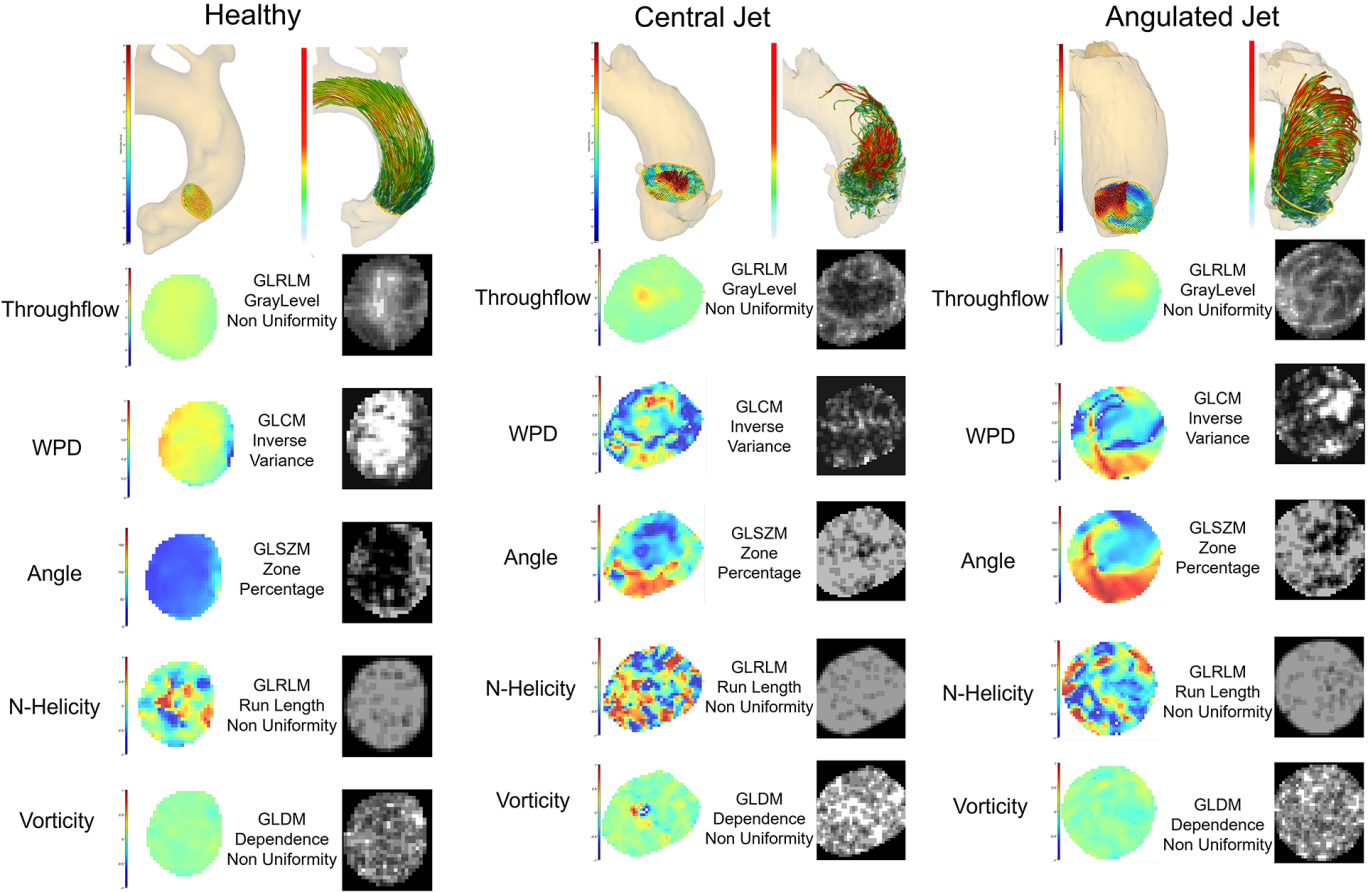
Radiomics feature analysis of the systolic cross-sectional flow profile in a healthy subject and two patients with different flow patterns caused by aortic valve stenosis. 3D renderings show the flow profile of the analysis plane A1.1 and the corresponding pathline visualization of the flow through this plane. The throughflow, WPD, angle, normalized helicity and vorticity feature maps represent a systolic timepoint. The corresponding radiomics maps were extracted by the pyradiomics library and display the feature, which was most reproducible in the inter-scanner comparison.

To test if radiomics signatures can be used to quantitatively describe flow profiles, we examine three cases, which are visually different to a human observer. Figure 6 shows three cases with different flow profiles caused by aortic stenosis (cohort 2) in comparison to a normal subject from cohort 3. The observable differences in the 3D vector visualization of the systolic cross-sectional flow result in clearly distinguishable feature distributions.

**Figure 7:**
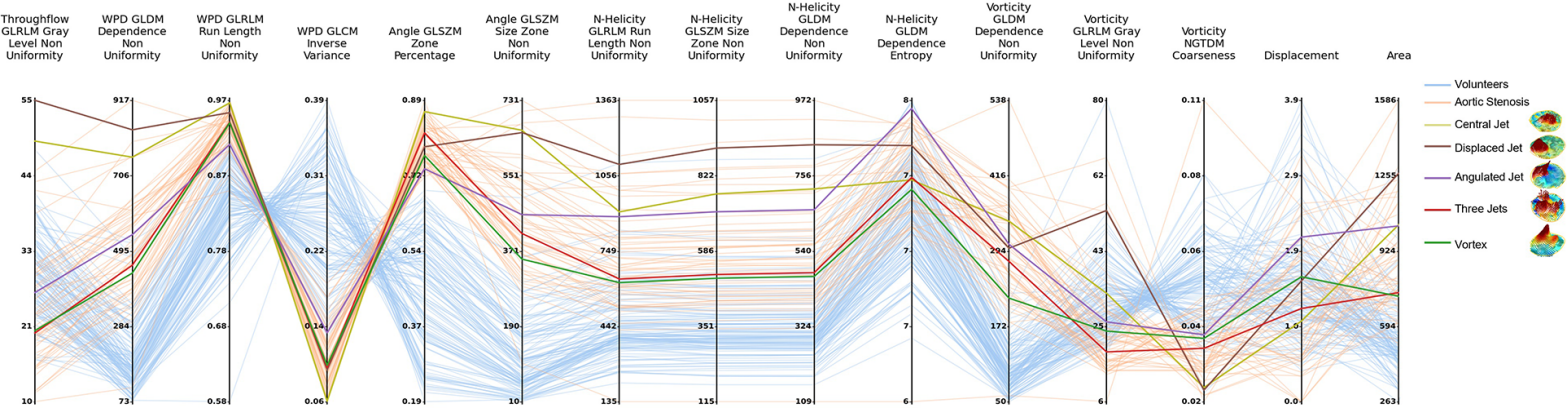
Parallel coordinates plot of selected radiomics features on data from all cases on slice A1.1. Features are extracted on the peak systolic phase with maximum volume flow rate. Examples of different central jet, displaced jet, angulated jet, three jets and vortex are highlighted.

The corresponding quantitative values of the radiomics features can be found in the supplementary material. A parallel coordinate plot visualization is shown in Figure 7. To provide an intuitive overview, we kept only at least moderately reproducible features, with visually different values for the selected patients. The patients are represented by lines in the plot. Cohort 2 (aortic valve stenosis) is represented by orange lines, blue lines represent cohort 3 (no valve disease). The colored lines display the selected cases with distinctly different flow profiles. The visualization reveals several features with no or little overlap between cohort 2 and 3 based on the distribution of the parameters wall parallelity degree (WPD), the flow angle, and the normalized helicity. The different flow profiles show individual feature combinations represented by different line patterns in the parallel coordinate plot.

## 4. Discussion

While the inter-observer agreement of radiomics features was excellent for 283 features, the inter-scanner agreement was only moderate in the best cases. Amongst these, only one feature was derived from the throughflow velocity map. This is in line with the findings by Demir et al., who found considerable differences in the velocity magnitudes measured with the different scanners (19). For wall parallelity degree, normalized helicity, and vorticity only higher-order features showed moderate agreement. For the direction-based angle map, which is independent of the velocity magnitude, the number of moderately reproducible features was considerably higher. Previous studies showed that spatial resolution and signal-to-noise ratio, slice thickness and field strength (27–30) have an impact on radiomics parameters. Especially in the context of 4D PC MRI, the selection of the velocity encoding can have an influence on the radiomics features. One example of a case in a healthy female volunteer scanned with different velocity encodings and thus in velocity-to-noise ratio is presented in the Figure S13 in the supplement.

In accordance with previous studies, we found sex and age-related differences in the aortic cross-sections (16, 31), which were also reflected in the radiomics features. These were accompanied by age-related differences in the patterns of flow directions, normalized helicity and vorticity, which were observed in patients with aortic valve stenoses as well. As shown in Figure 4, the average values of all features differed between subjects with and without aortic stenosis. This is in accordance with previous studies using parameters such as angle and normalized velocity to identify pathological flow patterns in patients with bicuspid aortic valves (32).

The classification test using the features with moderate reproducibility to distinguish between normal valves and aortic valve stenosis based on the systolic parameter maps of cross-section A1.1 in the ascending aorta (see Figure 6) show perfect results as shown in Figure 5. From the 51 features considered for the training of the classifier, the ones which describe homogeneity, and properties of homogeneous regions in the parameter maps of angle, normalized helicity and WPD were considered most important. Shape features had no significant influence. The underlying parameters have been suggested in previous publications (5, 16, 32). The standardized analysis of their distribution patterns however enable a more robust consideration of disease recognition.

In the assessment of different types of systolic flow profiles in the ascending aorta, the radiomics signatures showed different value ranges than the cohort without valve disease. Furthermore, there were clear differences in the wall parallelity degree features and angle patterns. It might therefore be possible to use radiomics signatures of flow for 4D PC MRI-based phenotyping in future studies. The explainability of the origin of the radiomics features allows for an advanced exploration of properties of detected phenotypes. Figure 6 links the radiomics signatures from the parallel coordinate plot in Figure 7 to the underlying feature and parameter maps as well as to the 3D position and flow visualization. The differences in the Gray Level Non Uniformity of the throughflow and the WPD maps clearly differentiate the compact jets from the other flow patterns. The Inverse Variance of the WPD map and the Zone Percentage of the Angle map order the flow profiles according to the amount of local differences in flow direction. The Non Uniformity features of N-Helicity maps separate the types of jets. Figure 6 shows the systolic N-Helicity maps for normal flow and two different jets. The maps differ in size of homogeneous regions, and the map with the smaller connected regions, which corresponds to a coarser flow profile, has a higher Non Uniformity feature value. The vorticity maps in Figure 6 display distribution patterns, which are relatively similar to those of the H-Helicity map, but the GLDM-based Dependence Non-Uniformity results provides a different order of jet types. The Coarseness feature based on the NGTDM separates the angulated and vortex flow from directed flow.

With recent developments in deep learning-based segmentation, the presented processing pipeline could be integrated into automatized and standardized analysis workflows to further seamlessly support image-based diagnostics and decision-making in clinical applications.

### 4.1. Limitations

Inter-observer analysis was only performed on a small number of healthy volunteers, and intra-observer and scan-rescan reproducibility was not assessed. However, based on the results of previous publications (17, 19), we assume that the radiomics feature agreement would be better than the inter-scanner results. The influence of different velocity encodings and thereby different VNR values could be further explored in future work. The datasets of cohorts 2 and 3 were acquired with different scanners, protocols and sequences than those represented in the Travelling colunteers study. While the spatial resolution was comparable in cohorts 2 and 3 to the Travelling-Volunteers-study, the temporal resolution was substantially higher in cohort 3. Furthermore, the velocity encoding in aortic stenosis patients with 350–600 cm/s was higher than 150–250 cm/s in the volunteer datasets. Due to the changes in the scan parameters we cannot be certain that the agreement of the derived radiomics features is comparable.

### 4.2. Conclusions and outlook

We introduced a concept for the calculation of radiomics signatures in order to quantitatively characterize flow profiles in the aorta. Radiomics features were selected based on their inter-scanner and inter-observer reproducibility and tested for the assessment of sex-, age- and disease-specific differences. We applied selected features to user-defined types of aortic flow profiles. The differences in the corresponding radiomics signatures suggest their utility in PC-MRI-based phenotyping and quantification of disease-related changes. With the new concept radiomics features could be used for an automatic assessment and comparable quantitiative description of hemodynamics properties, which mimics human visual analysis. This could enable the application in clinical studies as well as the definition of decision relevant radiomics signature ranges.

## Data Availability

The datasets generated and analyzed during the current study are not publicly available but are available from the corresponding author on reasonable request.

## Footnotes

^1^ https://pyradiomics.readthedocs.io/en/latest/
