# Supplementary material for: Radiomics-based aortic flow profile characterization with 4D phase-contrast MRI
Source: Front Cardiovasc Med. 2023 Apr 3;10:1102502. doi: 10.3389/fcvm.2023.1102502 (PMC10106758; doi:10.3389/fcvm.2023.1102502)
Supplement: Supplementary file 1 [file Datasheet1.pdf]

Supplementary Material

1 FIGURES

1.0.1 Inter Observer Reproducibilty

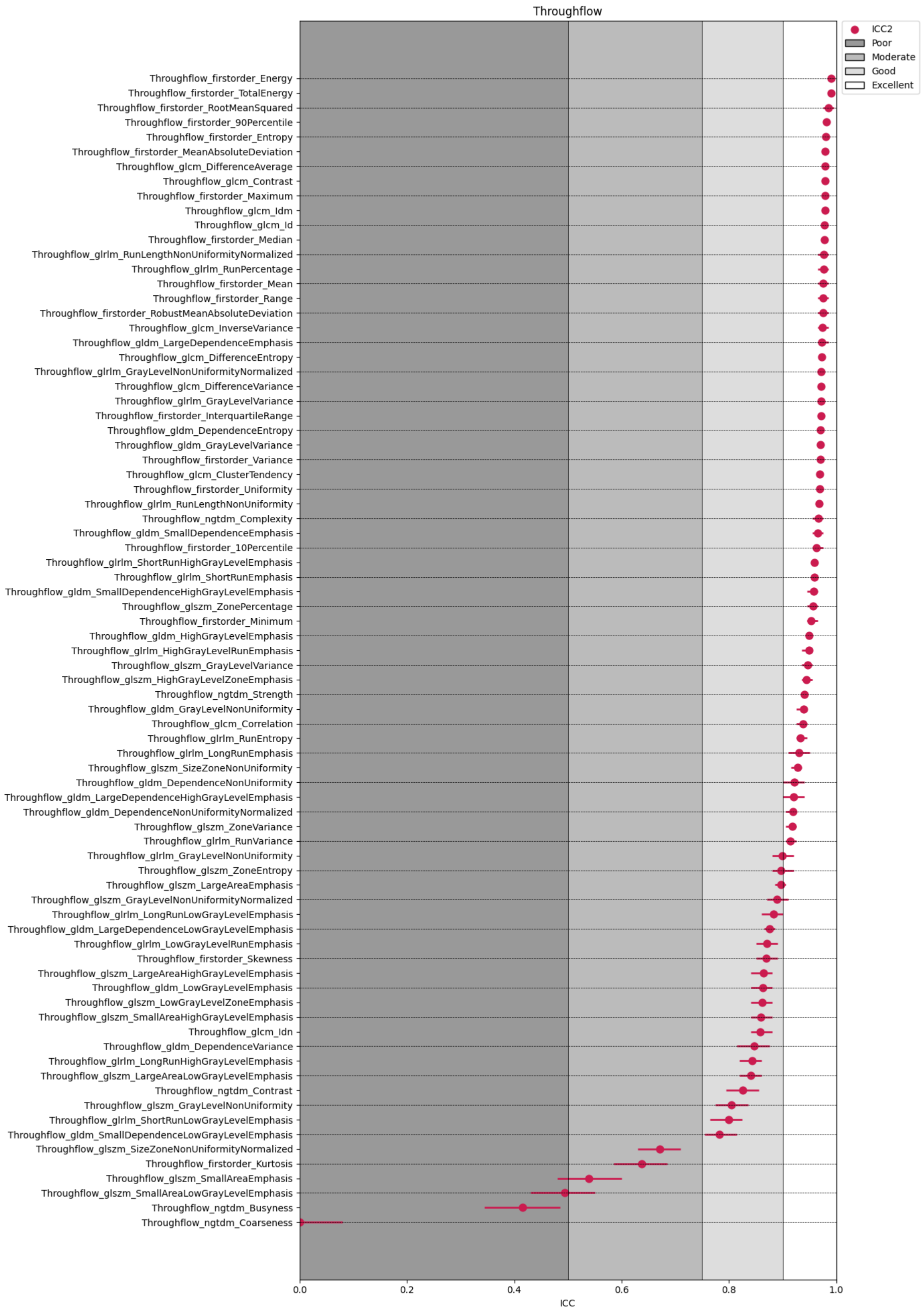

Figure S1. Inter-observer agreement of all features of throughflow features. Differences in shading indicate the reproducibility thresholds adopted from Koo and Li

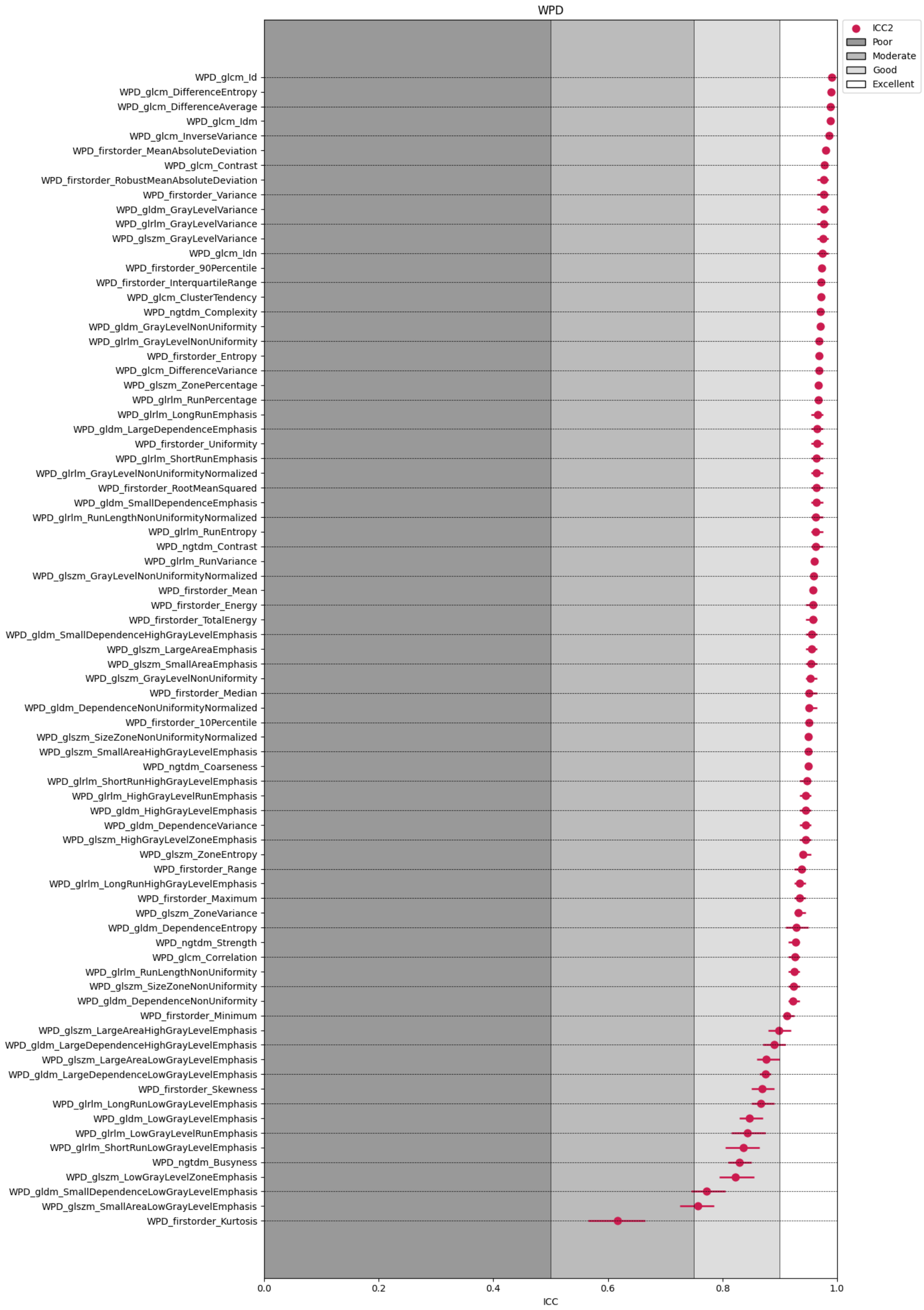

Figure S2. Inter-observer agreement of all features of WPD features. Differences in shading indicate the reproducibility thresholds adopted from Koo and Li

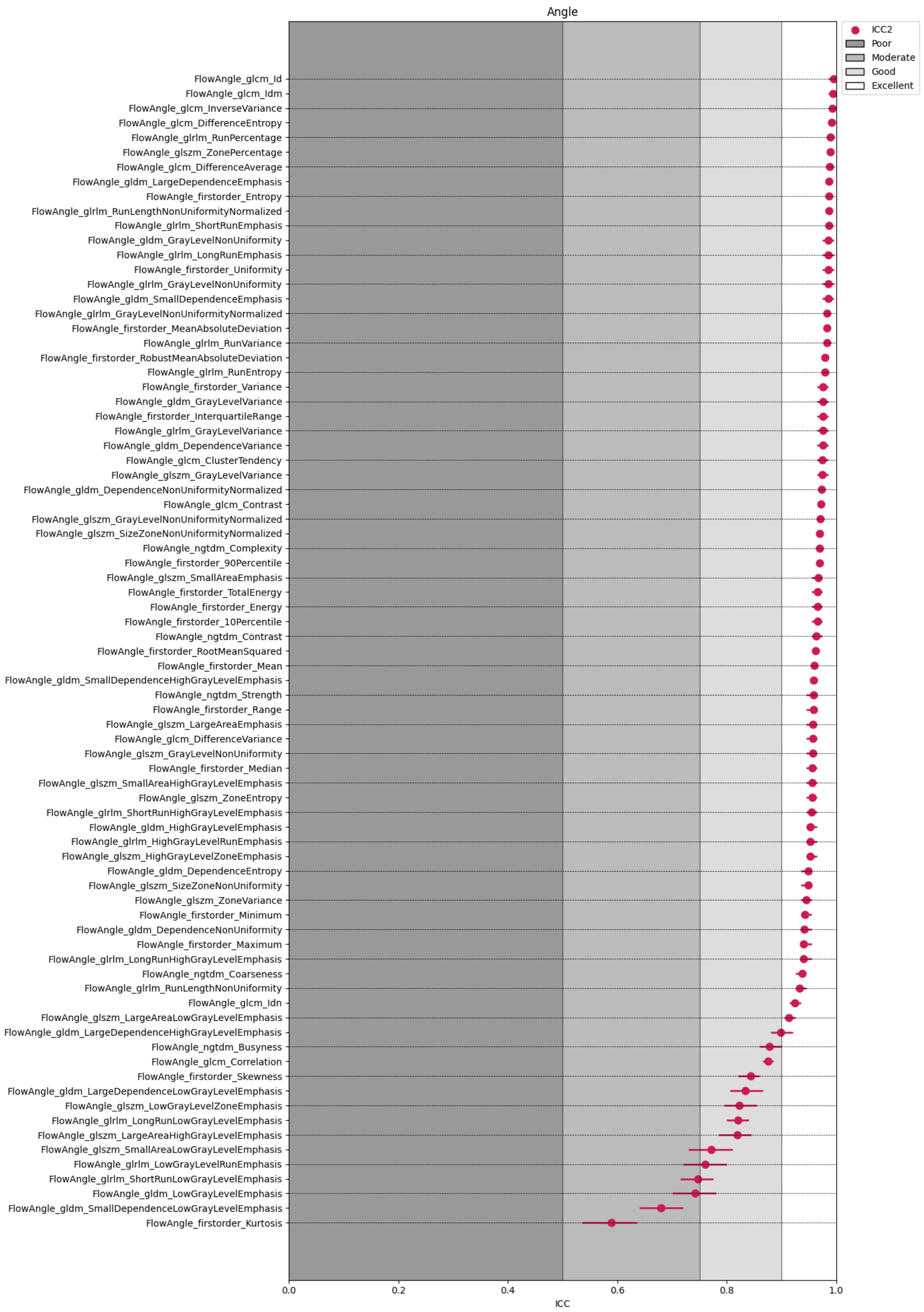

Figure S3. Inter-observer agreement of all features of angle features. Differences in shading indicate the reproducibility thresholds adopted from Koo and Li

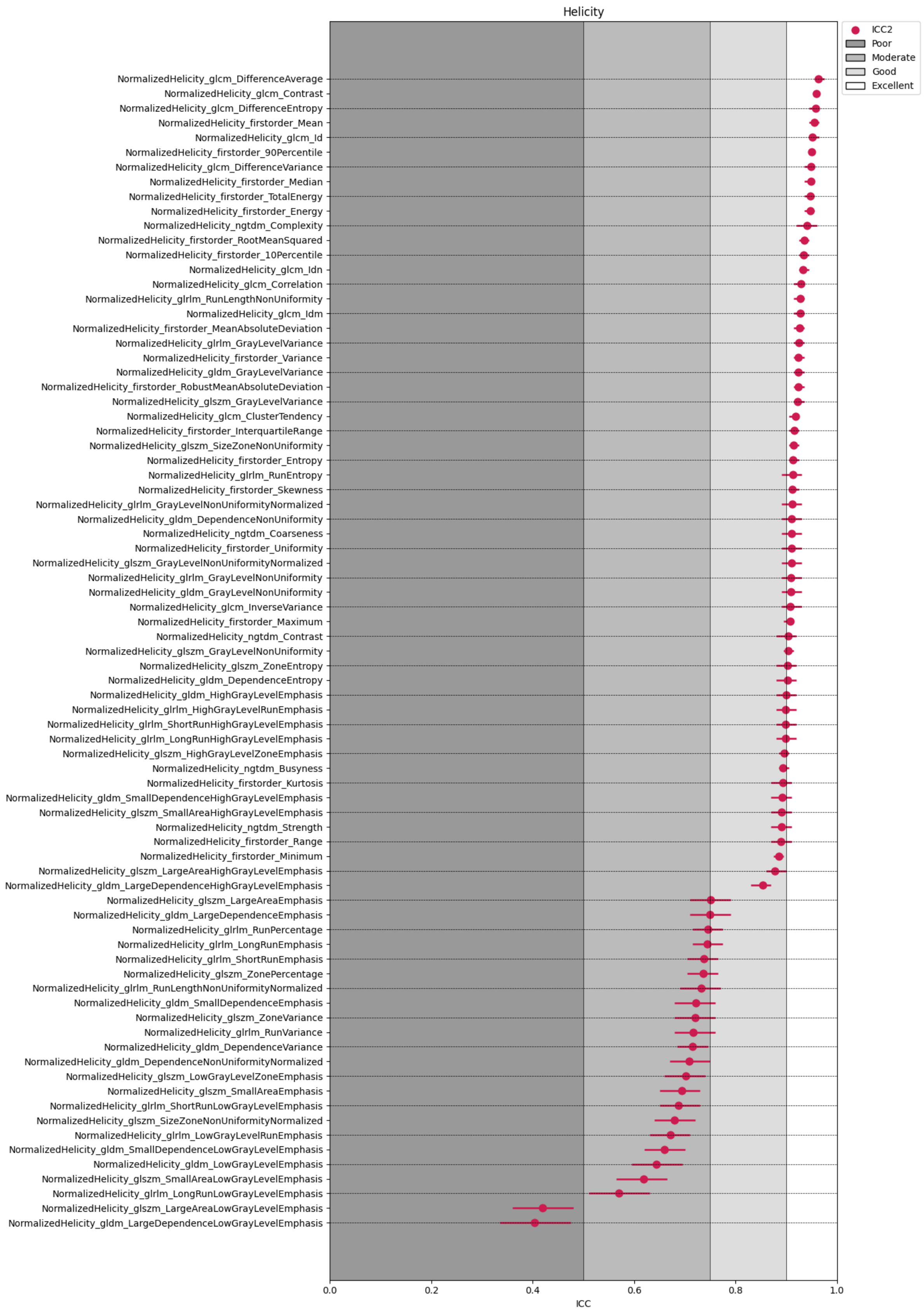

Figure S4. Inter-observer agreement of all features of helicity features. Differences in shading indicate the reproducibility thresholds adopted from Koo and Li

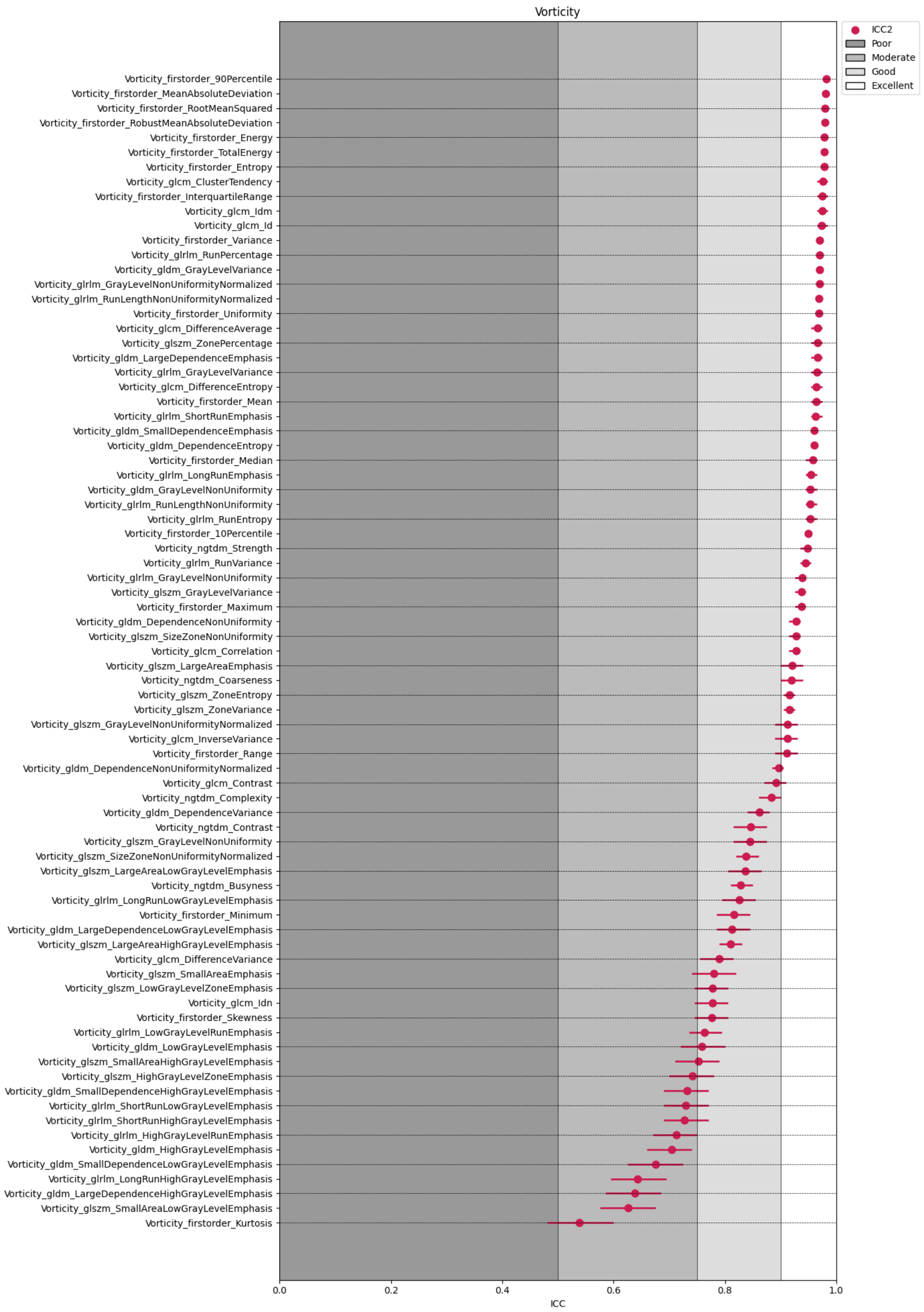

Figure S5. Inter-observer agreement of all features of vorticity features. Differences in shading indicate the reproducibility thresholds adopted from Koo and Li

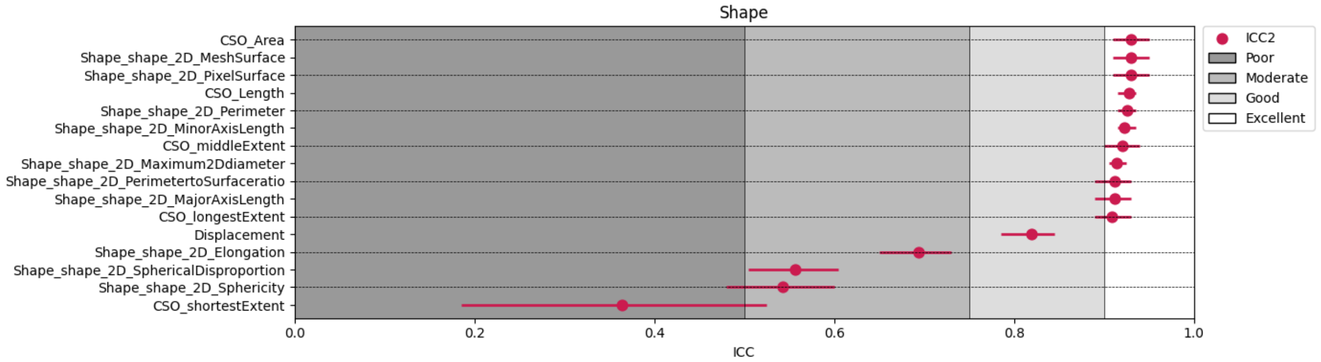

Figure S6. Inter-observer agreement of all features of shape features. Differences in shading indicate the reproducibility thresholds adopted from Koo and Li

1.0.2 Inter Scanner Reproducibilty

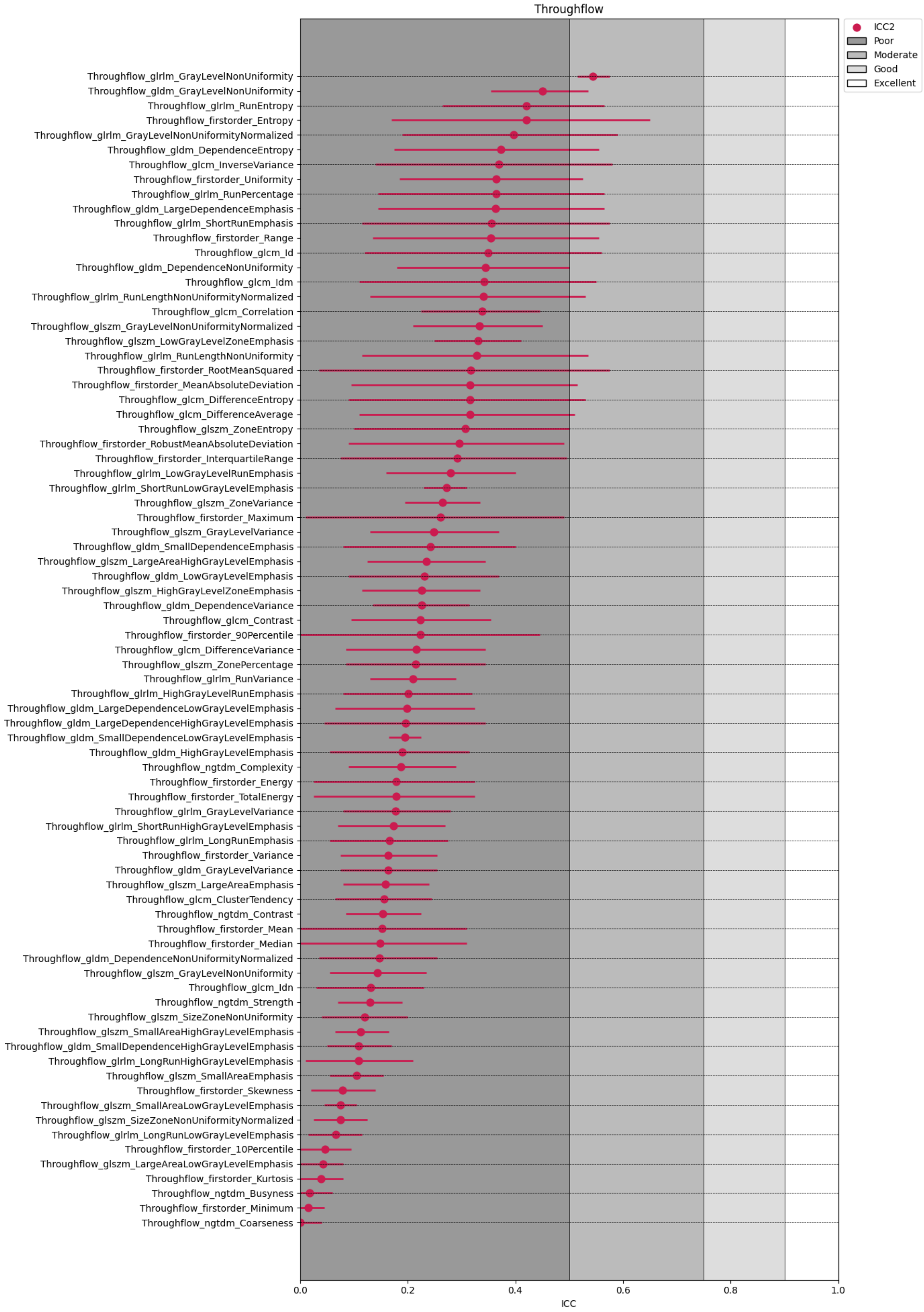

Figure S7. Inter-scanner agreement of all features of throughflow features. Differences in shading indicate the reproducibility thresholds adopted from Koo and Li

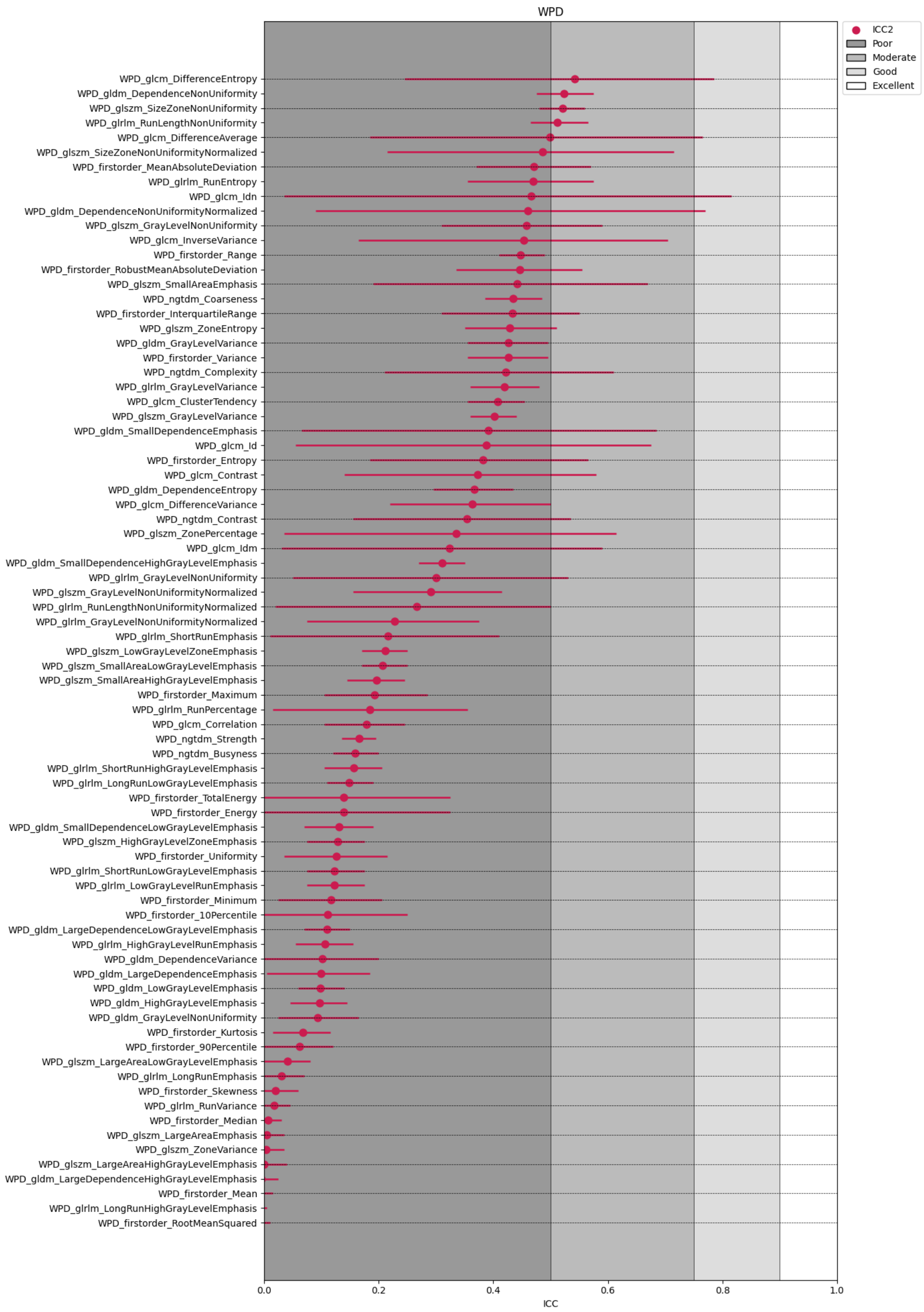

Figure S8. Inter-scanner agreement of all features of WPD features. Differences in shading indicate the reproducibility thresholds adopted from Koo and Li

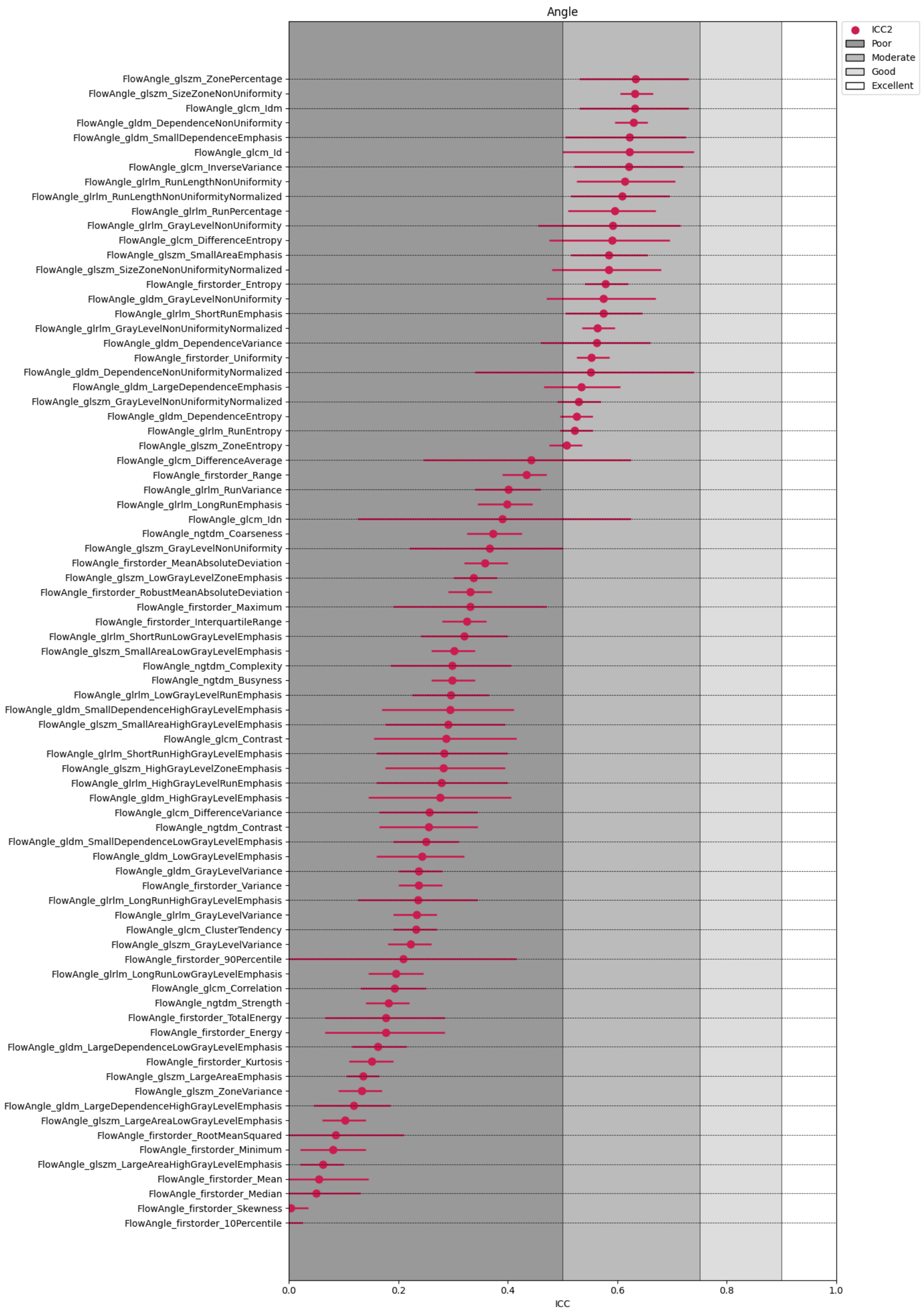

Figure S9. Inter-scanner agreement of all features of angle features. Differences in shading indicate the reproducibility thresholds adopted from Koo and Li

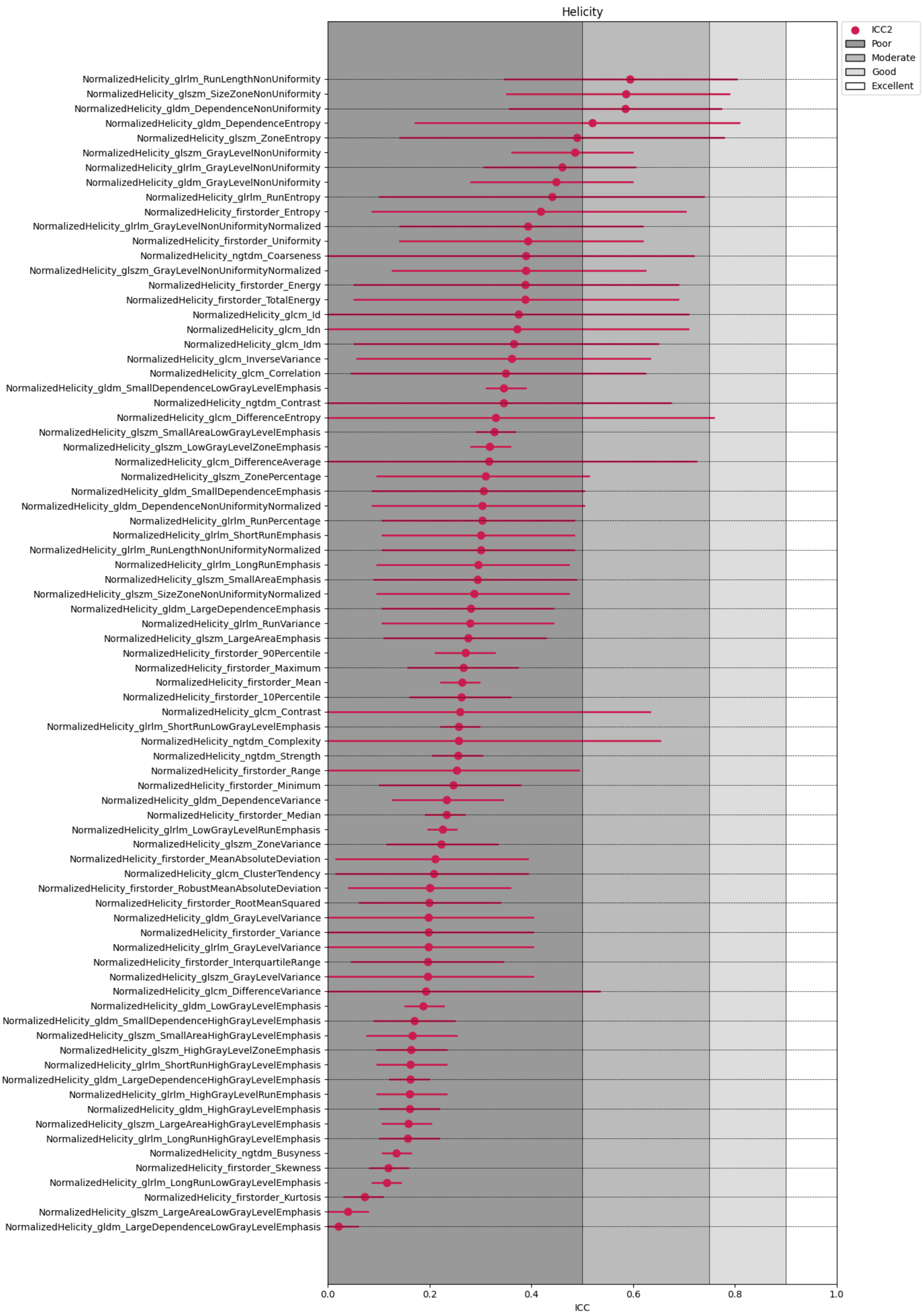

Figure S10. Inter-scanner agreement of all features of helicity features. Differences in shading indicate the reproducibility thresholds adopted from Koo and Li

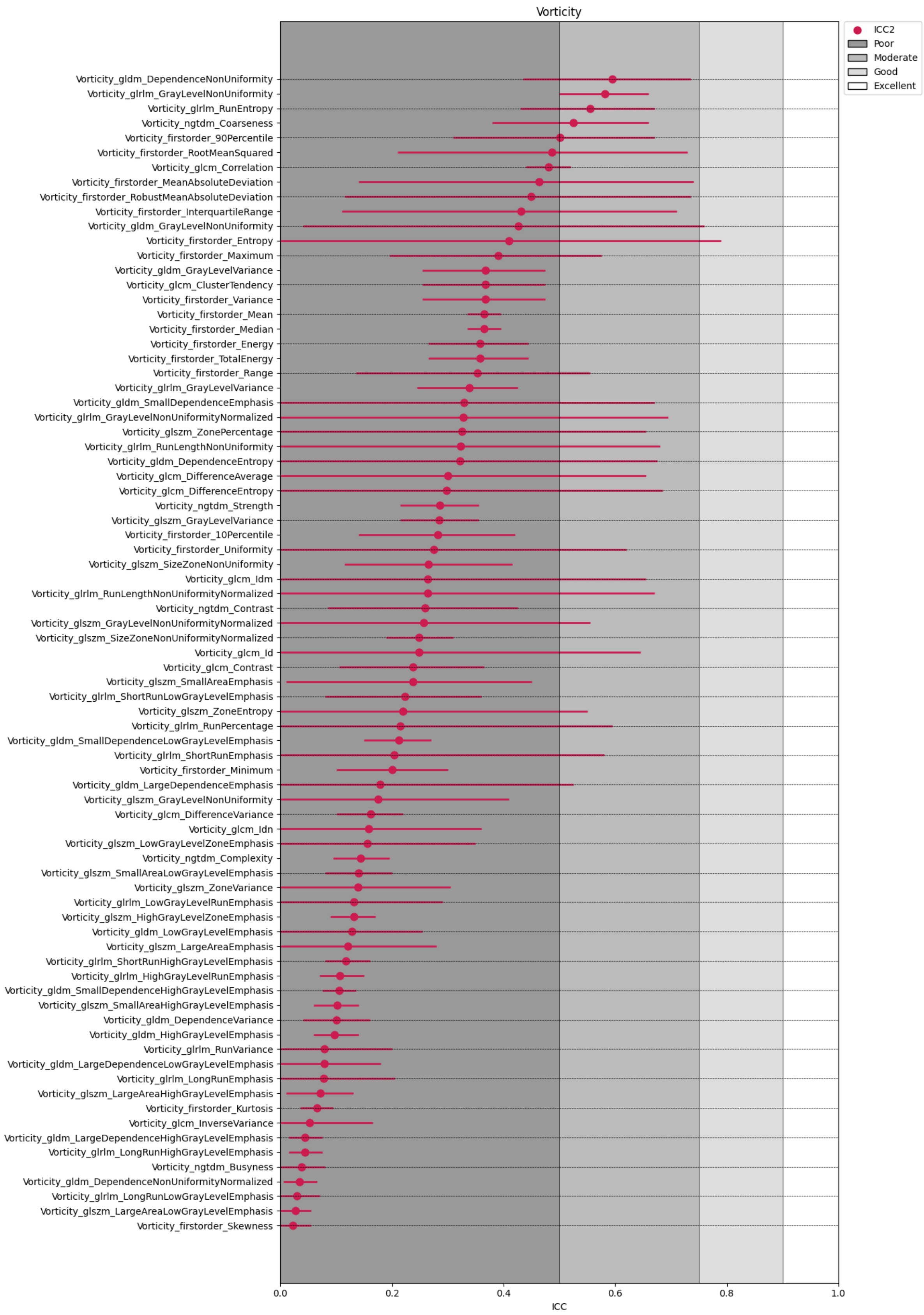

Figure S11. Inter-scanner agreement of all features of vorticity features. Differences in shading indicate the reproducibility thresholds adopted from Koo and Li

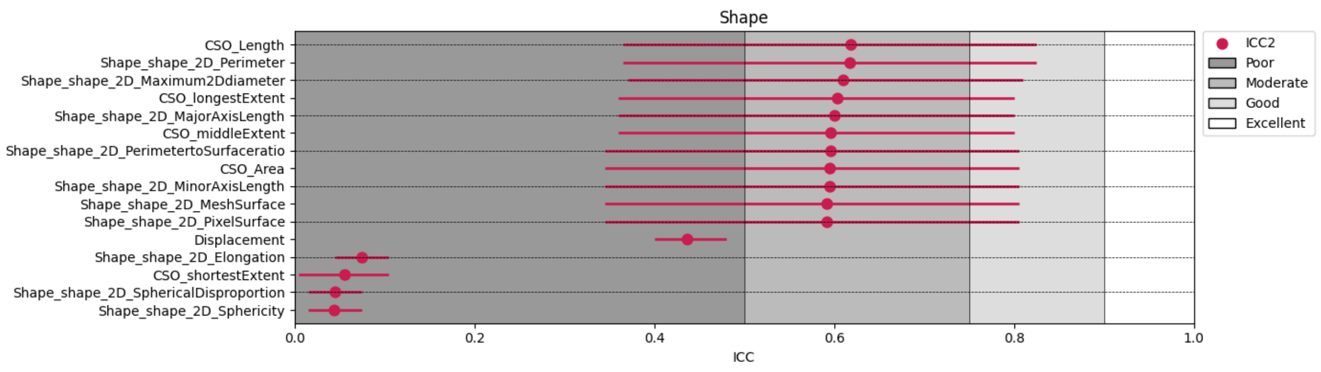

Figure S12. Inter-scanner agreement of all features of shape features. Differences in shading indicate the reproducibility thresholds adopted from Koo and Li.

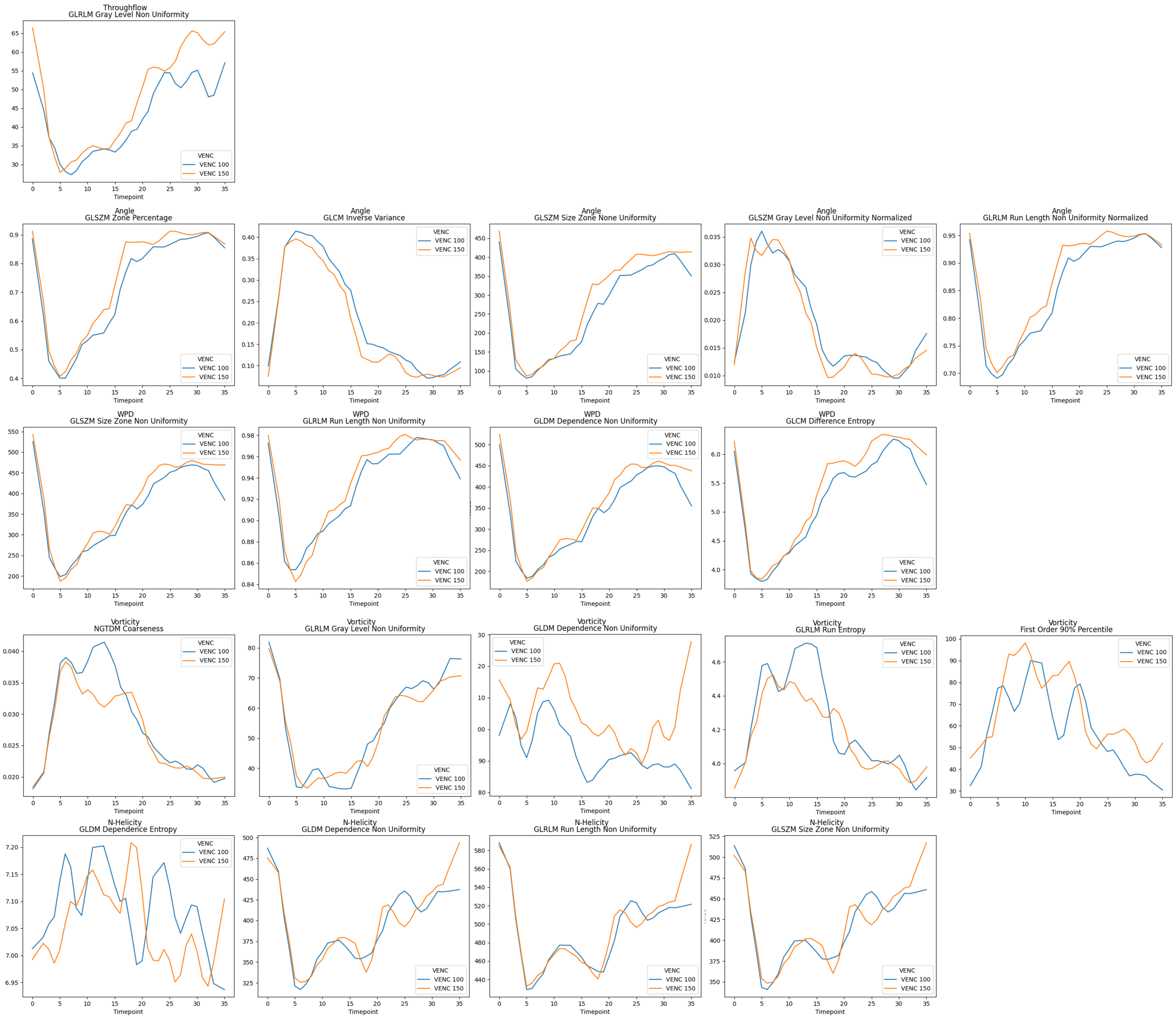

Figure S13. Influence of velocity to noise ratio (VNR) on radiomics parameters. Diagrams show radiomics feature curves extracted on an ascending aortic plane (A1.1) over the complete cardiac cycle. To analzye the influence of VNR on selected parameters, a healthy volunteer was scanned twice with a velocity encoding (VENC) of 100 cm/s and 150 cm/s without leaving the scanner.

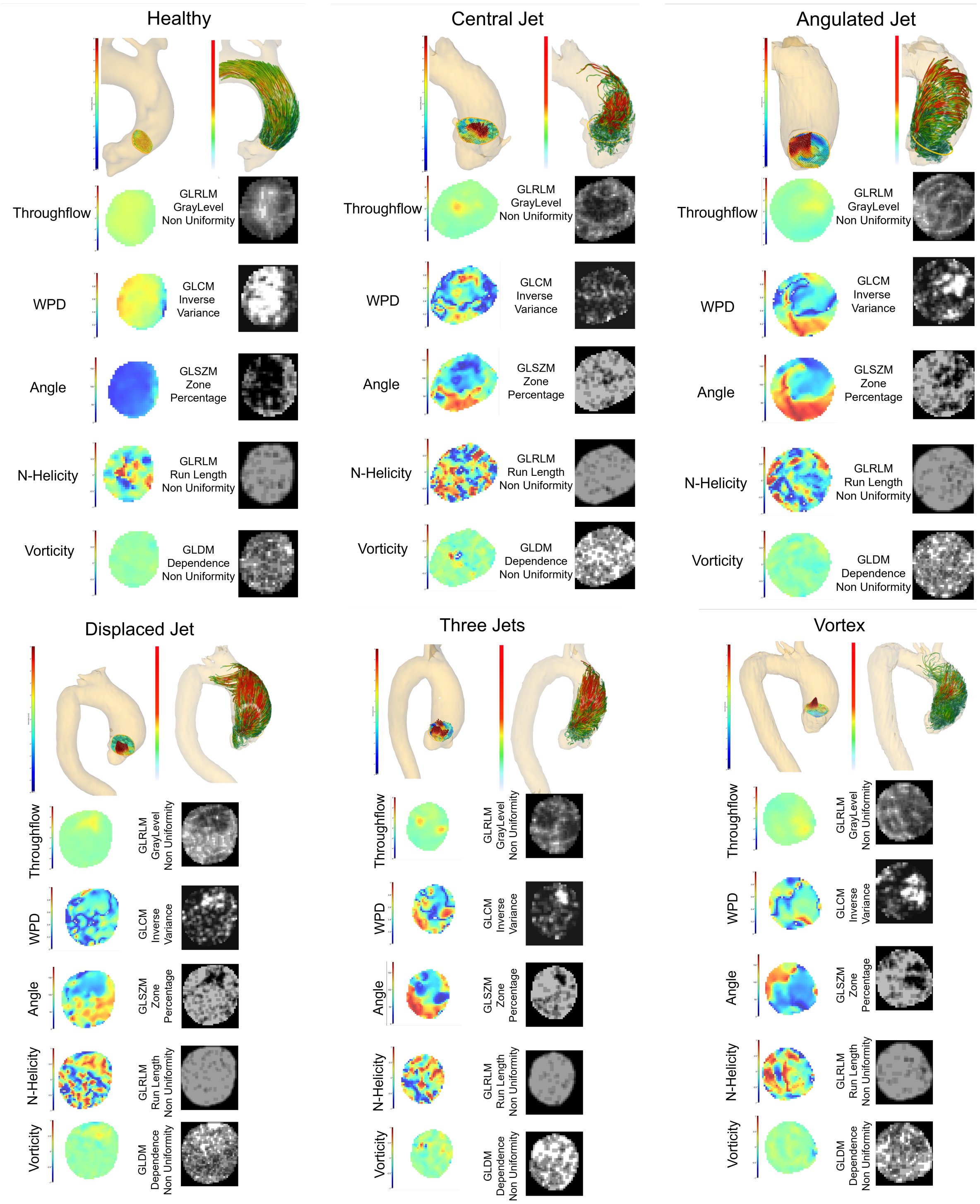

Figure S14. Radiomics feature analysis of the systolic cross-sectional flow profile in a healthy subject and five patients with different flow patterns caused by aortic valve stenosis. 3D renderings show the flow profile of the analysis plane A1.1 and the corresponding pathline visualization of the flow through this plane. The throughflow, WPD, angle, normalized helicity and vorticity feature maps represent a systolic timepoint. The corresponding radiomics maps were extracted by the pyradiomics library and display the feature, which was most reproducible in the inter-scanner comparison.
